# The Role of Cyclodextrins in the Design and Development of Triterpene-Based Therapeutic Agents

**DOI:** 10.3390/ijms23020736

**Published:** 2022-01-10

**Authors:** Alexandra Prodea, Alexandra Mioc, Christian Banciu, Cristina Trandafirescu, Andreea Milan, Roxana Racoviceanu, Roxana Ghiulai, Marius Mioc, Codruta Soica

**Affiliations:** 1 Department of Pharmaceutical Chemistry, Faculty of Pharmacy, Victor Babes University of Medicine and Pharmacy, 2nd Eftimie Murgu Sq., 300041 Timisoara, Romania; alexandra.ulici@umft.ro (A.P.); andreea.milan@umft.ro (A.M.); babuta.roxana@umft.ro (R.R.); roxana.ghiulai@umft.ro (R.G.); marius.mioc@umft.ro (M.M.); codrutasoica@umft.ro (C.S.); 2 Research Centre for Pharmaco-Toxicological Evaluation, “Victor Babes” University of Medicine and Pharmacy, Eftimie Murgu Sq., No. 2, 300041 Timisoara, Romania; alexandra.petrus@umft.ro; 3 Department of Anatomy, Physiology, Pathophysiology, Faculty of Pharmacy, Victor Babes University of Medicine and Pharmacy, 2nd Eftimie Murgu Sq., 300041 Timisoara, Romania; 4 Department of Internal Medicine IV, Faculty of Medicine, Victor Babes University of Medicine and Pharmacy, 2nd Eftimie Murgu Sq., 300041 Timisoara, Romania

**Keywords:** cyclodextrins, triterpenes, inclusion complexes, phytocompounds, cytotoxicity

## Abstract

Triterpenic compounds stand as a widely investigated class of natural compounds due to their remarkable therapeutic potential. However, their use is currently being hampered by their low solubility and, subsequently, bioavailability. In order to overcome this drawback and increase the therapeutic use of triterpenes, cyclodextrins have been introduced as water solubility enhancers; cyclodextrins are starch derivatives that possess hydrophobic internal cavities that can incorporate lipophilic molecules and exterior surfaces that can be subjected to various derivatizations in order to improve their biological behavior. This review aims to summarize the most recent achievements in terms of triterpene:cyclodextrin inclusion complexes and bioconjugates, emphasizing their practical applications including the development of new isolation and bioproduction protocols, the elucidation of their underlying mechanism of action, the optimization of triterpenes’ therapeutic effects and the development of new topical formulations.

## 1. Introduction

According to the World Health Organization (WHO) [1,2], chronic, non-transmittable conditions, such as cancer, hypertension, diabetes, stroke, chronic obstructive pulmonary disease, Alzheimer’s disease, renal abnormalities [3], etc., account for over 60% of deaths worldwide; the other 40% are represented by transmissible conditions such as lower tract respiratory pathologies, diarrheal diseases, tuberculosis and neonatal conditions. Despite the vast existing therapeutic arsenal, there are several diseases [4,5] without an efficient cure available as well as patients who do not respond to current therapies.

Plants or plant organs have been employed since ancestral times as treatment for various diseases, with mixtures of various medicinal plants being the primordial medicines used by humans. This approach has not undergone substantial changes over the years and today, according to WHO, around 80% of the earth’s population still mainly uses plant-derived remedies despite the massive advance in chemically synthetic drugs [6]. Nowadays, medicinal plants are extensively investigated for the development of new therapeutic agents due to their complex composition. However, current drug development requires the use of purified active compounds in order to ensure the reproducibility of the therapeutic outcome [7]. Secondary metabolites, such as triterpenes and their derivatives, are frequently used in drug development due to their proven biological activities with potential applications in the treatment of several diseases, including cancer [8].

Cyclodextrins (CDs) are compounds of natural origin, derived from starch and frequently used in the pharmaceutical field as drug carriers and excipients for the formulations of numerous bioactive compounds [9]. Their high biocompatibility and low toxicity has allowed for the development of several available formulations aimed for the topical, nasal, oral and parenteral delivery of active drugs, and even more formulations are currently undergoing the development process [10].

The literature regarding the use of cyclodextrins associated with triterpenic compounds is vast and complex; however, to the best of our knowledge, there are a limited number of reviews [11,12] dedicated to the applications of cyclodextrins-triterpenes inclusion complexes or conjugates in drug development. This paper aims to summarize the most important achievements made in the last decade in this field, emphasizing the main practical applications.

## 2. Triterpenic Compounds—Classification and Biological Activities

Triterpenes form a versatile class of compounds with over 30,000 representatives that have been isolated from both prokaryotic and eukaryotic cells, in particular from vegetal sources. The terms “triterpenes” and “triterpenoids” are both used to describe the same class of isoprene derivatives with 30 carbon atoms; however, the term “triterpene” generally refers to natural compounds isolated from plants, while “triterpenoids” indicate the compounds obtained through triterpenes’ natural degradation or semisynthetic structural modulation [13].

Triterpenes can be classified as acyclic and cyclic compounds, according to the presence of cycles in their structure; cyclic triterpenes are in turn classified according to the number of cycles in monocyclic, bicyclic, tricyclic, tetracyclic and pentacyclic compounds [14]. Of these, pentacyclic triterpenes are the most thoroughly investigated due to their multitude of biological actions such as anticancer, anti-inflammatory, antioxidant, antiviral and antidiabetic effects [15].

Studies conducted on pentacyclic triterpenes have shown their low absorption from plant products at the intestinal level [16] caused by their hydrophobic structure that significantly reduces their bioavailability and therapeutic effect [17]. Many formulation strategies have been developed to overcome this drawback, including the use of cyclodextrins, liposomes, nano-emulsions and nanoparticles [12]. Several biologically relevant triterpenic compounds included in CDs formulations in the last decade as well as their previously reported therapeutic effects are presented in Table 1. The previously and ongoing large body of work undertaken for the investigation of triterpenes has highlighted several biological activities related to these compounds, including anticancer, anti-inflammatory, antioxidant and antimicrobial effects, while new biological activities including antidiabetic, antiosteoporotic and neuroprotective activities are currently under evaluation.

**Figure 1:**
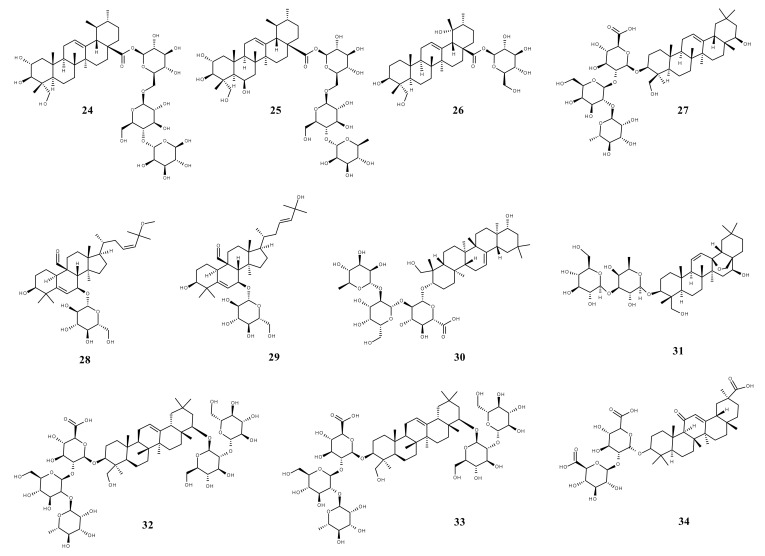
The chemical structure of several triterpenic saponins: Asiaticoside (**24**), Madecassoside (**25**), Pedunculoside (**26**), Azukisaponin V (**27**), Momordicoside K (**28**), Momordicoside L (**29**), Soyasaponin I (**30**), Saikosaponin-D (**31**), Bersimoside I (**32**), Bersimoside II (**33**) and Glycyrrhizic acid (**34**).

Triterpenic saponins are natural amphiphilic compounds that possess a lipophilic aglycon with triterpenic structure and a hydrophilic component consisting of two to five sugar units in their structure; the two components are linked by one or two glycosidic bonds [60]. The saponin derivatives discussed in this paper are depicted in Figure 1.

## 3. Cyclodextrins—Classification and Physicochemical Properties

Cyclodextrins (CDs) are a class of natural compounds, obtained through the enzymatic hydrolysis of starch, intensely used in recent decades in the development of new therapeutic, cosmetic and nutraceutical formulations. Their structure consists of several glucose units connected by α-(1–4)-glycosidic bonds, which induce the formation of a hydrophobic internal cavity and a hydrophilic outer surface, in the form of a truncated cone. The internal cavity of CDs allows for the incorporation of hydrophobic compounds with a suitable molecular size [61].

**Figure 2:**
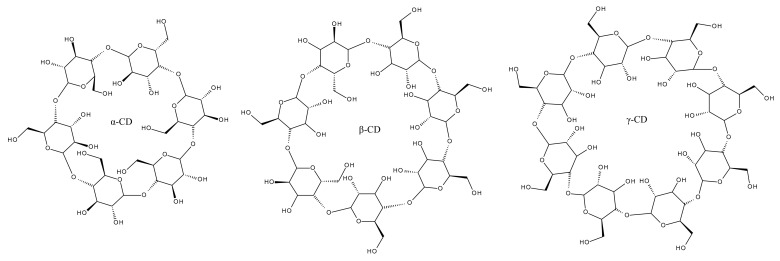
Structures of native cyclodextrins.

CDs can be divided into two main classes: natural CDs, namely α, β and γ-CD (Figure 2), and semisynthetic CDs obtained through the derivatization of the hydroxyl residues using various groups. Depending on the groups grafted to the CDs surface, the obtained derivatives possess several advantages such as increased inclusion capacity for certain compounds, superior water solubility (HP-β-CD) or lipophilicity (Methyl-β-CD) [62].

When administered orally, native cyclodextrins are absorbed in small amounts from the gastrointestinal tract and are eliminated unchanged through the urinary tract, resulting in low toxicity at low-to-moderate doses. Following parenteral administration, β-CD induces nephrotoxicity due to its low water solubility, which hampers its parenteral use. As a rule, hydrophilic CDs derivatives are generally well tolerated after oral and parenteral administration, producing only minor side effects such as diarrhea; in contrast, lipophilic CDs have been shown to have toxic effects due to their increased gastrointestinal absorption; therefore, their administration being currently limited [63].

Because CDs are versatile structures, industrially obtained through simple processes that allow for the synthesis of products with superior analytical purity, they have been successfully used in multiple fields, including the pharmaceutical practice in order to obtain complexes with plant extracts [64], essential oils [65] and also pure compounds of natural or synthetic origin [66]. The incorporation of lipophilic compounds into CDs is the most frequent approach to improve the solubility and bioavailability of such compounds [67].

The inclusion complexes formed by CDs with various drugs possess several advantages over the original compound, such as the following: improved solubility (Table 2), stability and bioavailability; the reduction of unpleasant organoleptic characteristics; the prevention of interactions with other drugs or excipients; and the modified or controlled release of the compound [68]. Recently, a new strategy designed to improve drug bioavailability and biological activity is the synthesis of new CDs’ active compound bioconjugates by the chemical modulation of the hydroxyl groups situated on the CDs’ exterior surface [69].

## 4. Inclusion Complexes with Triterpenes—Methods of Preparation and Characterization

The literature describes numerous techniques that can be used to form inclusion complexes, the selected procedure depending firstly on the available equipment (i.e., mortar and pestle, grinder, mill) and secondly on the process parameters such as temperature, water amount in the environment, mixing time and drying conditions [76]. The preparation methods of inclusion complexes can be classified into three main categories according to the components’ state of aggregation at the time of preparation: solid state (physical mixing and grinding), semi-solid state (kneading, slurry-complexation) and liquid state methods (coprecipitation, spray drying, lyophilization) [77].

Several preparation methods were used in the development of CD inclusion complexes with triterpenic compounds or plant extracts containing triterpenic compounds, as displayed in Table 3 and Table 4. The most commonly used complexation method is the low-cost and simple kneading procedure, consisting in the treatment of suitable mixed amounts of the guest molecule and CD with a small quantity of water and/or ethanol; at the laboratory scale, this method can be easily applied using a mortar and pestle, while at a larger industrial scale, extruders are required [78]. However, the most advantageous methods for the solvent removal used in the liquid and semi-solid state preparation of CDs are the lyophilization and spray-drying methods. Lyophilization consists of mixing the CD and guest compound in a liquid medium followed by a freeze-drying process and can be successfully employed for thermosensitive guests with a good yield of complexation. The spray-drying method can also be applied to a liquid mixture of CD and guest compound by using a spray-dryer; because temperatures of 50–70 °C can be reached inside the equipment, this method is only appropriate for thermostable compounds [79].

**Figure 3:**
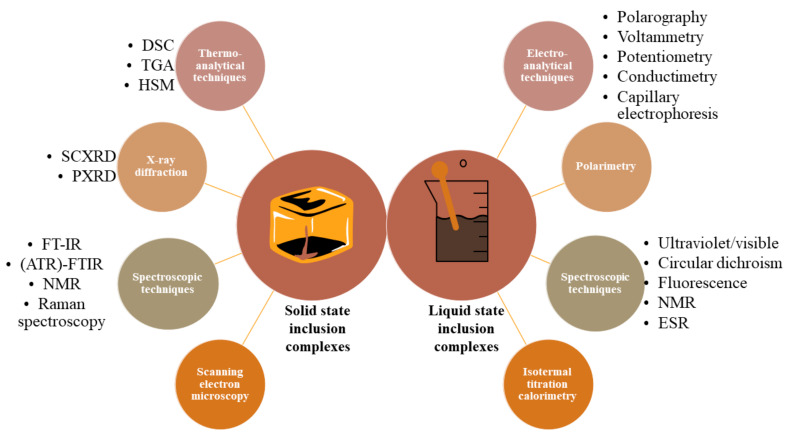
An overview of the methods used to characterize inclusion complexes in solid and liquid state (differential scanning calorimetry (DSC), thermogravimetric analysis (TGA), hot-stage microscopy (HSM), single-crystal X-ray diffraction (SCXRD), powder X-ray diffraction (PXRD), Fourier-transform infrared (FT-IR) spectroscopy, attenuated total reflectance (ATR)-FTIR spectroscopy, nuclear magnetic resonance (NMR) and electron spin resonance (ESR)).

The validation of the inclusion phenomenon together with the determination of the complex stability constant, Kc, represent important steps in the development and characterization of new inclusion complexes with therapeutic value. A multitude of analytical methods can be employed in the characterization of such complexes, mainly depending on the state of aggregation, solid or liquid, of the analyzed sample. Recently, two well-documented papers regarding the techniques used in the characterization of solid [96] and liquid (aqueous) [97] CD inclusion complexes were published; Figure 3 displays an overview of the most frequently used analytical methods in the assessment of inclusion complexes.

A method that is gaining popularity in this field is represented by Density Functional Theory (DFT), a computational method used to predict the structure of the inclusion complex by enabling the calculation of Gibbs free energy, enthalpy and entropy [98]. This method offers a more efficient alternative to determine the optimal CD for a particular triterpenic compound by saving valuable material resources and time.

As an example, the affinity capillary electrophoresis was used for the determination of the Kc values for three inclusion complexes (1:1 molar ratio) between HP-γ-CD and several hydrosoluble Bet derivatives: betulin 3,28-diphthalate (DPhB), betulin 3,28-disuccinate (DScB), betulin 3,28-disulfate (DSB) and betulin 3-acetate-28-sulfate (ASB), respectively; the results showed superior water solubility and stability to the inclusion complexes formed with pure Bet, as quantified by the increased log Kc values as shown in Table 5 [24,25].

An interesting strategy to highly improve the solubility of triterpenic compounds is the use of *Nicotiana tabacum* (Solanaceae) and *Catharanthus roseus* (Apocynaceae) cells as biotransformation centers; thus, three BoA derivatives with therapeutic potential were metabolized into compounds with superior water solubility. However, inclusion complexes with HP-γ-CD had to be obtained in order to solubilize the initial compounds in the hydrophilic culture medium (50).

**Figure 4:**
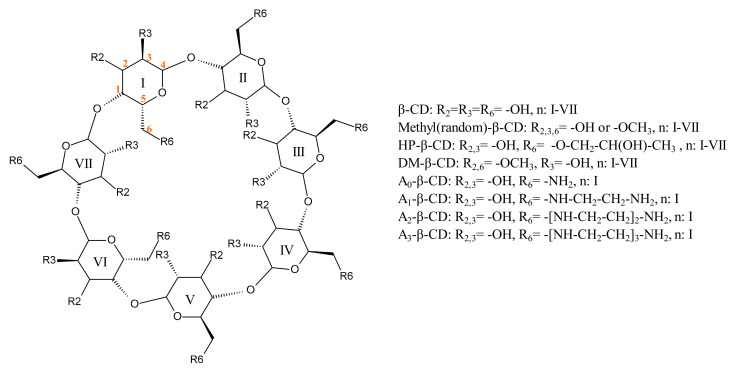
Chemical structure of β-CD derivatives: “n” denominates the glucopyranose ring/rings where the indicated functional groups are grafted.

There are various types of CDs available for the entrapment of triterpenic compounds, including β-CD derivatives (Figure 4); finding the optimal CD for the incorporation of a particular biologically active triterpene is essential in the design and development of new effective therapeutic alternatives. Following the complexation of compound K, a triterpenoid isolated from *Panax ginseng*, with β-CD and γ-CD, respectively, it was found that γ-CD is the most suitable host for its incorporation, leading to much higher solubility and stability enhancement compared to β-CD [100]. Ramos et al. studied the complexes formed by aescin, a mixture of triterpenic saponins extracted from the seeds of *Aesculus hippocastanum* (Hippocastanaceae), where β-aescin is the main component and represents a mixture of aescin Ia and Ib; its inclusion in β-CD and γ-CD was hypothesized to increase the bioavailability of the active compounds. Aescin formed 1:1 and 1:2 complexes with γ-CD and β-CD, respectively, thus suggesting that the larger γ-CD cavity may better accommodate the aescin molecule [101].

A number of factors can influence the process of formation as well as the stability of triterpene inclusion complexes with various CDs. The study conducted by Lopez-Miranda et al. showed that for OA and MA, complexes formed with native β-CD, exhibit superior stability to those obtained with HP-β-CD, γ-CD and HP-γ-CD, respectively. Additionally, the temperature increase during preparation (t = 65 °C) together with an alkaline pH (pH = 9), which favors acid ionization, thus promoting bond formation with the CD hydrophobic cavity, induce an increased stability of the formed complexes [83].

The inclusion of a compound inside CDs is not necessarily the optimal formulation for all compounds and cannot guarantee an increased bioavailability. For instance, complexation of koetjapic acid with HP-β-CD in various molar ratios (1:2, 1:4, 1:6) only slightly improved the water solubility of the compound; by contrast, the conversion of the compound in its potassium salt led to a 65-fold higher aqueous solubility compared to the CD complexation (30). Modified CDs were synthesized in order to improve the stability of various inclusion complexes; however, they can also exert a negative influence on the complex formation and stability. The presence of the hydroxypropyl group is not always beneficial in the formation of triterpene complexes, as in the case of BA and BoA, which were not able to form inclusion complexes with HP-β-CD, probably due to the reduced size of the β-CD hydrophobic cavity caused by the hydroxypropyl group [102].

## 5. Applications of Triterpene: Cyclodextrin Inclusion Complexes in the Pharmaceutical Field

**Figure 5:**
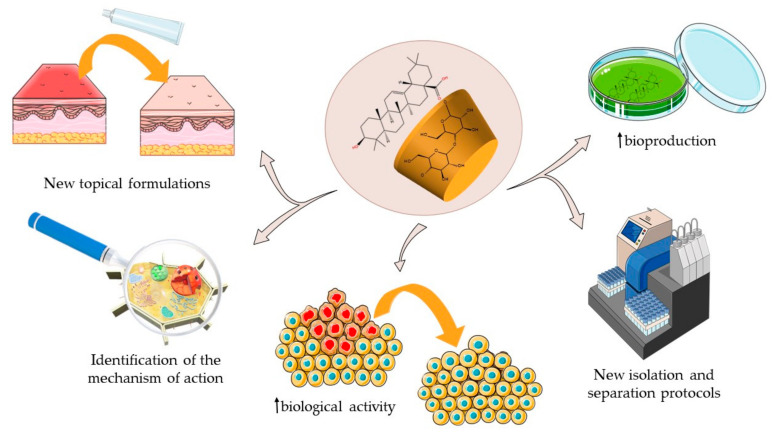
An overview of the applications of CD–triterpenic compounds’ inclusion complexes and conjugates in the pharmaceutical field.

The main goal in pharmaceutical research is the development of effective and well-tolerated drugs; a current strategy in this field is the use of natural-origin compounds, such as triterpenes, as scaffolds for the development of new therapeutic alternatives [103]. Many research groups are currently working towards this goal [104], starting from the early stages of drug development—identification, isolation, structural optimization and in vitro/in vivo testing of the active drug—to the more advanced stages of establishing the underlying mechanism of action and developing clinical trials. CDs have been successfully employed in several stages of this process for the development of new therapeutic agents with triterpenic structure as illustrated below (Figure 5).

### 5.1. Development of New Protocols for the Separation and Isolation of Triterpenic Compounds

Plants contain a great diversity of compounds with different physicochemical properties and therapeutic potential [105]. The separation of compounds with similar structures or properties from the total extract is generally difficult and requires a complex working protocol [106]; therefore, the development of new, fast and efficient separation techniques continues to be highly relevant.

The development of a HPLC protocol allowed for the separation of the two triterpenic isomers, OA and UA, that differ from one another by the position of a single methyl group, which makes their separation very difficult. The addition of γ-CD to the mobile phase increased the solubility of the triterpenic acids in the eluent and reduced the column retention time, thus increasing their separation selectivity. Following the use of α-CD and β-CD, the separation of the compounds did not occur, presumably due to either the small size of the CD cavity that does not allow for the incorporation of such molecules or the low stability of the formed complexes, which prevents their detection in solution [107]. A significant issue is the selection of native or semisynthetic CDs to be used as additives to the mobile phase in order to achieve the best separation parameters; Wang et al. [108,109] assessed the effectiveness of several CDs, both native and semisynthetic, in the separation of five triterpenic compounds with anti-inflammatory and antioxidant properties extracted from *Centella asiatica* (Apiaceae). The authors developed a HPLC protocol with isocratic dilution where various CDs were added to the mobile phase, which allowed for the simultaneous separation in a time-effective manner of two triterpenic acids, madecassic and asiatic acid, and three triterpenic glycosides, asiaticoside B, madecassoside and asiaticoside. The significant difference in polarity between triterpenic acids and triterpene glycosides makes their separation highly difficult; the addition of CDs in the mobile phase induced the formation of inclusion complexes, which significantly altered triterpenes’ retention time. However, the nature of the cyclodextrin was revealed as essential: while γ-CD was able to achieve the separation of all five triterpenoids, α-CD, β-CD and β-CD derivatives were effective only for the elution of triterpene glycosides, which can form hydrogen bonds with the glucose residues of the cyclodextrins. The triterpenic acids could be eluated only in the presence of γ-CD, which not only enabled their separation, but also sharply decreased their retention times presumably due to the formation of inclusion complexes. These findings were corroborated with the determination of the apparent formation constants, which depend on the nature of both triterpene and cyclodextrin; for the triterpene glycosides, larger values were recorded for native CDs compared to their hydrophilic semisynthetic derivatives presumably due to the presence of substituent groups on the CD’s chain, which might prevent the formation of inclusion complexes. For the two triterpenic acids, large formation constants were reported with γ-CD due to their fitted accommodation inside the CD’s cavity and the formation of a stable inclusion complex. Capillary electrophoresis (CE) is a separation technique conducted in a capillary tube and based on the differentiated migration of the ionic charged components of a mixture under the influence of an electric field [110]. This method exhibits a series of advantages that support its wide use, such as time effectiveness, low solvent consumption and high-resolution separation even for stereoisomers [111]. Using the CE method, the simultaneous separation of two pairs of diastereomers triterpenic saponins from *Trifolium alexandrinum*, namely soyasaponin I, azukisaponin V, bersimoside I and bersimoside II, was achieved by using a dual mechanism that involves the addition of β-CD to the borate buffer solution [112]; saponins were included in the CD cavity, thus achieving a very effective separation characterized by high resolution, repeatability and detection limits.

The CE method also allowed for the simultaneous identification of three isomers with pentacyclic triterpenic structure, BA, OA and UA, from the *Forsythia suspensa* fruit, a plant commonly used in traditional Chinese medicine, by adding CDs to the buffer solution in order to act as isomer modifiers. However, the study reported that of all tested CDs, namely α-CD, β-CD, γ-CD and methyl-β-CD, the separation of the three isomers was achieved only in the presence of β-CD; moreover, the resolution highly depended on the β-CD concentration as well as the stability constants of the resulting inclusion complexes [113].

High-speed countercurrent chromatography (HSCCC) is a separation method based on the principle of liquid–liquid partition, which has been used in multiple separations of natural compounds since 1898 [114]. β-CD was added to the mobile phase in an HSCCC protocol in order to separate two isomeric triterpenes, MA and corosolic acid, extracted from the leaves of *Eriobotrya japonica* (Rosaceae) and differentiated by the position of a single methyl group. The addition of β-CD allowed for the recovery of the two triterpenes in a yield of over 90% from the plant extract [115]. Another study reported the high-resolution separation by HSCCC of the two isomeric triterpenes, OA and UA, extracted from *Eriobotrya japonica*, by adding HP-β-CD to the mobile phase in order to form inclusion complexes with the two compounds, respectively. [116].

Molecularly Imprinted Solid-Phase Extraction is a technique that consists in creating a polymer template for compounds of interest from a complex mixture, such as plant extracts. This is a highly selective separation method that can be customized for the isolation of a unique compound of interest [117]. The method was successfully used to isolate glycyrrhizic acid (GA) from a licorice roots aqueous extract by using a polymeric matrix formed of methacrylic acid and a modified CD, bismethacryloyl-β-CD (BM-β-CD), both acting as functional monomers [118]. The authors reported that the aromatic rings of the phytocompound were accommodated inside the CD monomer, leaving the polar carboxylic group outside the cavity; the addition of the second monomer, methacrylic acid, lead to the formation of hydrogen bonds with the free carboxylic moiety. Overall, the polymer matrix exhibited high-adsorption ability and fast binding kinetics for the phytocompound, thus enabling its extraction from licorice roots with optimized yields in an aqueous environment.

Collectively, multiple techniques including HPLC, CE, HSCC and Molecularly Imprinted Solid-Phase Extraction can be successfully employed in the isolation and separation of triterpenic compounds from vegetal mixtures; the addition of various CDs, both native and semisynthetic, may lead to improved yields in a time-efficient manner and may allow for the separation of isomeric compounds, a goal difficult to achieve by conventional procedures. The successful outcome of the applied analytical protocol greatly depends on several parameters including the nature and concentration of both active compounds and cyclodextrins. Future research should focus on the feasibility of applying these techniques on a larger scale in order to contribute to the development of pharmaceutical analysis.

### 5.2. Increasing the Bioproduction of Triterpenic Compounds

Nowadays, one can notice an increasing interest in the development of new sustainable and environment-friendly production methods of valuable secondary metabolites from plants and modified microorganisms that can be used as new therapeutic alternatives [119]. Some methods involve the use of elicitors, substances that can stimulate the biosynthesis of certain secondary metabolites. CDs are considered true elicitors as a result of their ability to facilitate the biosynthesis of bioactive compounds such as silymarin [120], trans-resveratrol [121] and taxol [122]; they also can favor the excretion of hydrophobic compounds, such as triterpenic compounds, through cell membranes.

A culture of *Saccharomyces cerevisiae* has been genetically modified in order to produce an antitumor triterpenoid derived from ganoderic acid, namely ganoderic acid 3-hydroxy-lanosta-8,24-dien-26-oic acid (GAN-HLDOA). The yeast cells are not naturally able to excrete the newly synthesized triterpenoid; this drawback was solved by the addition of cyclodextrins to the culture medium, which were able to capture the triterpenoid through the pores of the cell membrane without penetrating and influencing the intracellular environment. Of the five tested cyclodextrins, the three native cyclodextrins, α, β and γ, and the two β-CD derivatives, 2-hydroxypropyl-b-cyclodextrin (HP-β-CD) and 2,6-dimethyl-b-cyclodextrin (DM-β-CD), the β-CD derivatives achieved superior results probably due to the larger size of their hydrophobic cavities as well as to the positive influence of the hydroxypropyl and dimethyl groups on the stability of the formed complexes [123]. Similar results that emphasize the superior effect of semisynthetic CDs over native CDs on the bioproduction of triterpenic compounds were obtained from the evaluation of a genetically optimized culture of *Saccharomyces cerevisiae* able to produce β-hydroxy-amyrin. In brief, the three native CDs and the semisynthetic methyl-β-CD derivative, respectively, were added to the culture medium, in order to facilitate the triterpenoid excretion from the yeast cells without altering their cell membranes. As previously reported for GAN-HLDOA, the highest amount of β-hydroxy-amyrin was obtained by using methyl-β-CD, which exhibited optimal size and lipophilicity of the inner cavity, thus allowing for the formation of a stable inclusion complex [124].

Taraxasterol and taraxerol are two oleane-type pentacyclic triterpenes with well-recognized anticancer properties; it was reported that β-CD proved to be more effective than methyl jasmonate as a stimulating agent for the production of the two triterpenes by a plant cell culture obtained from the root of *Taraxacum officinale* (Asteraceae), thus providing a suitable alternative to the current production methods [125]. These findings were endorsed by several other studies; as such, Briceño et al. reported the superior elicitor properties of CDs (Cavasol^®^) over methyl jasmonate for the production of taraxasterol by a *Solanum lycopersicum* (Solanaceae) cell culture [126], while Commault et al. developed a method to isolate triterpenic compounds with therapeutic potential from *Chlamydomonas reinhardtii* (Chlamydomonadaceae), a single-cell green alga, by adding methyl jasmonate and methyl-β-CD, respectively, to the culture medium; the authors reported the accumulation of cycloartenol in the intracellular environment as a result of methyl-β-CD addition. In addition, the β-CD derivative did not influence the growth of algae cells; moreover, when methyl-β-CD and methyl jasmonate were added together to the culture medium, the CD simultaneously reduced the side effects produced by methyl jasmonate, such as increased oxidative stress, inhibition of cell growth and alteration of the photosynthesis process [127].

Collectively, although plant sources are currently considered the etalon of renewable resources for now, the use of genetically modified bacteria and plant cell cultures has started to gain the attention of the scientific community due to optimized time effectiveness and reproducibility. However, this area is rather poorly explored at the moment for triterpenic compounds; therefore, future research should be carried out in order to develop methods for the biosynthesis of other triterpenic compounds, in particular those in high demand for the development of new therapeutic alternatives such as BA and OA.

### 5.3. Increasing the Therapeutic Effect of Triterpenic Compounds

CDs are usually considered inert excipients that enhance the biological activities of associated compounds by increasing their water solubility and bioavailability and protecting them from the degrading effects exerted by external factors such as oxygen, heat and visible and ultra-violet light; all these mechanisms increase the quantity of compounds that reach their biologic target, thus increasing their therapeutic effects (Table 6 and Table 7) [128].

A frequently mentioned hypothesis regarding the activity of medicinal plants is that total or partial vegetal extracts exert superior biological effects to individual compounds [129] due to the synergism of action between the various mixed compounds. This hypothesis was validated for the *Hypericum perforatum* (Hypericaceae) extract [130] and also tested for numerous medicinal plants [131,132], including plants containing triterpenic compounds.

As such, the synergism of action was tested for mistletoe, which contains two classes of compounds with proven cytotoxic action, namely lipophilic triterpenic acids (OA and BA) and hydrophilic lectins. Due to the different solubilities of these compounds, in order to assess the presence of their in vitro synergistic effects, the incorporation of the triterpenic acids into an aqueous solution was conducted by using 2-HP-β-CD as a solubility enhancer. The cytotoxicity of the mistletoe extracts, both simple (containing either triterpenes or lectins, respectively) and combined (containing a mixture of triterpenes and lectins in the presence of HP-β-CD) was tested on three cell lines, NALM-6 (Acute Lymphoblastic Leukemia) [90], HL-60 (Acute Myelogenous Leukemia) [91] and TC-71 (Ewing’s Sarcoma) [133]; when compared to simple extracts, all studies reported superior synergistic cytotoxic effects for the combined active compounds, the combination being enabled by the presence of the hydrophilic CD, which allowed for the water solubilization of the lipophilic triterpenes.

In a similar study, OA was combined with its isomer, UA, in order to assess their synergistic in vitro and in vivo cytotoxic activity against melanoma as well as the influence of cyclodextrin complexation on the cytotoxicity of both individual compounds as well as their combination; the results validated the occurrence of synergistic effects between the two acids and a slight improvement of cytotoxicity in the presence of HP-γ-CD, presumably due to its ability to increase the aqueous solubility of the two triterpenic compounds, [86] thus endorsing the previously reported study in terms of using CDs as solubility enhancers. The cytotoxicity assessment of several cyclodextrin inclusion complexes of OA and UA on melanoma cells identified 2-HP-β-CD and 2-HP-γ-CD as the optimal CDs for the incorporation of OA and UA, respectively, with the resulting complexes displaying increased cytotoxicity as well [85]. By contrast, a study that evaluated the cytotoxicity of a mistletoe extract rich in OA against two types of melanoma cells revealed that cyclodextrin complexation of the extract with 2-HP-β-CD did not achieve improved cytotoxicity [92]; therefore, the authors concluded that the increased stability of an inclusion complex cannot always be correlated with enhanced biological activity. However, the literature study emphasizes that, in most cases, the presence of CDs is beneficial for the increase of biological effects; as such, by including a mixture of α-amyrin and β-amyrin, two triterpenes with anti-inflammatory properties isolated from *Protium heptaphyllum* (Burseraceae), in β-CD and HP-β-CD, respectively, complexes with superior anti-inflammatory activity that reduced the cellular proliferation of J774 macrophages were obtained [87].

The inclusion of saikosaponin-d in HP-β-CD using various molar ratios (1:1, 1:5 and 1:10 phytocompound:cyclodextrin) increased its water solubility up to 1074-fold, simultaneously improving its cytotoxicity against the skin cancer cell line HSC-1; the 1:5 complex was revealed as the most effective in increasing both the solubility and cytotoxicity of the phytocompound [134].

The inclusion complex of BA, extracted from the juice of *Ziziphus jujuba* (Rhamnaceae) with β-CD revealed superior cytotoxic effects against breast cancer cells (MCF-7) compared to pure BA [82]. A much larger increase in cytotoxicity increase was, however, noticed for the inclusion complex of BA with a modified semi-synthetic γ-CD, (octakis-[6-deoxy-6-(2-sulfanyl ethane sulfonic acid)]-γ-cyclodextrin) when tested against melanoma, both in vitro, on cell cultures, and in vivo, on murine models; the effect can be attributed to the very high stability of the inclusion complex achieved due to the hydrophobic chains attached to the CD ring, which greatly contributed to the entrapment of the guest molecule. In addition, the semisynthetic γ-CD derivative displayed significantly higher water solubility compared to native CDs and led to highly aqueous soluble complexes, thus allowing for higher concentrations of BA to reach its target [81]. Therefore, the selection of the most suitable CD in terms of water solubility and complexation capability is a very important step in the development of CD-based drug carriers. In light of these findings, the development of new semisynthetic CDs, which display superior properties compared to natural CDs, represents a great opportunity for the pharmaceutical research. As such, Ren et al. developed, characterized and used amino-appended-β-CDs (Figure 4) to enhance the cytotoxicity of OA in vitro against three cancer cell lines, HepG2 (Hepatocellular Carcinoma), HT29 (adenocarcinoma) and HCT116 (adenocarcinoma), as well as one non-cancer cell line, MRC-5 (human fetal lung fibroblast). Among the evaluated complexes, $A_{0}$-β-CD was selected as a potential candidate for the development of a new therapeutic agent, with $IC_{50}$ values ranging from 0.67 to 2.06 µM against the cancer cell lines tested. Simultaneously, all of the evaluated inclusion complexes proved to be less cytotoxic against the non-cancerous MRC-5 cell line compared to pure OA, thus indicating higher selectivity and the improvement of the phytocompound’s safety profile [84].

GA is the aglycone of glycyrrhizin, a phytocompound extracted from the licorice root with anti-inflammatory and hepatoprotective effects; its triterpenic nature leads to a diminished oral bioavailability and biological activity. This drawback was overcome by the inclusion into HP-γ-CD, which proved to be more efficient in vivo in a rat model than the singular components in reducing the intestinal lesions produced by indomethacin, a non-steroidal anti-inflammatory drug known for its severe gastrointestinal side effects. The identified underlying mechanism consists in the suppression of several pro-inflammatory markers, namely tumor necrosis factor alpha (TNF-α), interleukin-1 beta (IL-1β) and interleukin-6 (IL-6) [135]. A distinct research group achieved the improvement the anticancer effect of GA, coupled the inclusion complex formed between GA and β-CD with a new type of fluorescent nanomaterials, quantum dots, in order to increase GA cytotoxicity and selectivity against HepG2 (hepatocellular carcinoma), HeLa (cervical cancer) and ECV-304 (urinary bladder carcinoma) cell lines. The formulations proved to be more effective against the HepG2 cell line compared to GA against the cell lines, where it induced apoptosis in a dose- and time-dependent manner through an alteration in the ROS-mediated pathway [136], thus showing that CDs can be successfully used in combination with other carriers for the enhancement of triterpenic compounds’ therapeutic properties.

**Table 6:** In vitro activity of triterpene:cyclodextrin inclusion complexes against cancer cell lines.

	Cyclodextrin Type	Type of Formulation	Cell Line Type	In Vitro Cell Model	In Vitro Antiproliferative Results for Extracts/Triterpenic Compounds	In Vitro Antiproliferative Results for Inclusion Complexes/Conjugates	Time of Incubation	References
		Plant Extracts						
A Viscum album extract containing Oleanolic acid (69.4%) and Betulinic acid (6.9%)	HP-β-CD	complex	Murine melanoma	B16.F10	$IC_{50}$ ≈ 13.7 µg/mL (OA in DMSO)	$IC_{50}$ ≈ 16.8 µg/ml	20 h	[92]
			Acute Lymphoblastic Leukemia	NALM-6	Inhibition ≈ 40% (Viscum extract)	Inhibition ≈ 80%	24 h	[90]
			Leukemia	U937	Inhibition ≈ 20% (Viscum extract)	Inhibition ≈ 42%	18 h	[91]
				HL-60	Inhibition ≈ 32% (Viscum extract)	Inhibition ≈ 90%		
			Ewings Sarcoma	TC-71	Inhibition ≈ 78% (Viscum extract)	Inhibition ≈ 80%	24 h	[133]
				MHH-ES-1	Inhibition ≈ 45% (Viscum extract)	Inhibition ≈ 50%		
Triterpenes								
BA	HP-β-CD	complex	Human breast cancer	MCF-7	$IC_{50}$ ≈ 25.3 µM	$IC_{50}$ = 11.6 µM	24 h	[82]
	octakis-[6-deoxy-6-(2-sulfanyl ethanesulfonic acid)] -γ-cyclodextrin		Murine melanoma	metastatic B164A5	Inhibition ≈ 42.1%	Inhibition ≈ 57.7%	72 h	[81]
				non-metastatic B164A5	Inhibition ≈ 38.2%	Inhibition ≈ 49.7%	72 h	
UA	HP-β-CD (1:2)	complex	Melanoma	A375	$IC_{50}$ ≈ 68.2 µM	$IC_{50}$ ≈ 51.7 µM	48 h	[85]
			Murine melanoma	B16 4A5	$IC_{50}$ ≈ 43.6 µM	$IC_{50}$ ≈ 40.9 µM		
			Melanoma	SK-Mel 2	$IC_{50}$ ≈ 58.4 µM	-		
	HP-γ-CD (1:2)			A375	$IC_{50}$ ≈ 68.2 µM	$IC_{50}$ ≈ 31.4 µM		
			Murine melanoma	B16 4A5	$IC_{50}$ ≈ 43.6 µM	$IC_{50}$ ≈ 16.1 µM		
			Melanoma	SK-Mel 2	$IC_{50}$ ≈ 58.4 µM	$IC_{50}$ ≈ 9.3 µM		
OA	HP-β-CD (1:2)	complex		A375	Inhibition ≈ 50%	-	4 h	[84]
			Murine melanoma	B16 4A5	Inhibition ≈ 20.7%	Inhibition ≈ 48.7%		
			Melanoma	SK-Mel 2	Inhibition ≈ 5%	-		
	HP-γ-CD (1:2)			A375	Inhibition ≈ 50%	-		
			Murine melanoma	B16 4A5	Inhibition ≈ 20.7%	Inhibition ≈ 45.7%		
			Melanoma	SK-Mel 2	Inhibition ≈ 5%	-		
OA	$A_{0}$-β-CD	conjugate	Hepatocellular Carcinoma	HepG2	$IC_{50}$ ≈ 66.3 µM	$IC_{50}$ ≈ 0.7 µM	4 h	[84]
			Adenocarcinoma	HT29	$IC_{50}$ ≈ 37.5 µM	$IC_{50}$ ≈ 2.0 µM		
				HCT116	$IC_{50}$ ≈ 27.9 µM	$IC_{50}$ ≈ 0.9 µM		
	$A_{1}$-β-CD		Hepatocellular Carcinoma	HepG2	$IC_{50}$ ≈ 66.3 µM	$IC_{50}$ ≈ 12.4 µM		
			Adenocarcinoma	HT29	$IC_{50}$ ≈ 37.5 µM	$IC_{50}$ ≈ 18.6 µM		
				HCT116	$IC_{50}$ ≈ 27.9 µM	$IC_{50}$ ≈ 11.4 µM		
	$A_{2}$-β-CD		Hepatocellular Carcinoma	HepG2	$IC_{50}$ ≈ 66.3 µM	$IC_{50}$ ≈ 23.0 µM		
			Adenocarcinoma	HT29	$IC_{50}$ ≈ 37.5 µM	$IC_{50}$ ≈ 18.9 µM		
				HCT116	$IC_{50}$ ≈ 27.9 µM	$IC_{50}$ ≈ 9.6 µM		
	$A_{3}$-β-CD		Hepatocellular Carcinoma	HepG2	$IC_{50}$ ≈ 66.3 µM	$IC_{50}$ ≈ 26.2 µM		
			Adenocarcinoma	HT29	$IC_{50}$ ≈ 37.5 µM	$IC_{50}$ ≈ 22.4 µM		
				HCT116	$IC_{50}$ ≈ 27.9 µM	$IC_{50}$ ≈ 21.7 µM		
Triterpenic saponins								
GA	β-CD	Complex, coupled with functionalized quantum dots	Hepatocellular Carcinoma	HepG2	$IC_{50}$ ≈ 423.2 µM	$IC_{50}$ ≈ 0.9–1.7 µM	48 h	[136]
			Cervical Cancer	HeLa	$IC_{50}$ ≈ 556.4 µM	$IC_{50}$ ≈ 1.8–2.9 µM		
			Urinary bladder carcinoma	ECV-304	$IC_{50}$ ≈ 673.6 µM	$IC_{50}$ ≈ 1.9–3.6 µM		
Saikosaponin-d	HP-β-CD (1:1)	complex	Squamous Cell Carcinoma	HSC-1	Inhibition ≈ 45%	Inhibition ≈ 50%	8 h	[134]
					Inhibition ≈ 65%	Inhibition ≈ 75%	24 h	
	HP-β-CD (1:5)				Inhibition ≈ 45%	Inhibition ≈ 40%	8 h	
					Inhibition ≈ 65%	Inhibition ≈ 60%	24 h	
	HP-β-CD (1:10)				Inhibition ≈ 45%	Inhibition ≈ 60%	8 h	
					Inhibition ≈ 65%	Inhibition ≈ 75%	24 h	

The binding of triterpenes to the surface of CDs using a triazole linker may increase the stability and water solubility of the resulting conjugates, as shown by Xiao et al., who linked a series of triterpenic acids, namely BA, OA, echinocystic acid (EA) and UA to native β-CD and methyl-β-CD using a 1,2,3-triazole moiety; bioconjugates with superior water solubility compared to the single compounds were obtained. The linkers were prepared in situ by a click chemistry reaction between the 6-azide-6-deoxy-β-CD or 6-azide-6-deoxyper-O-methylated-β-CD and alkynyl-functionalized triterpenic acids. Subsequently, the anti-entry action against the HCV virus of these bioconjugates was evaluated on four cell lines, namely HeLa (cervical cancer), HepG2 (hepatocellular carcinoma), MDCK (Madin–Darby canine kidney) and 293T (human kidney). The per-O-methylated β-CD–pentacyclic triterpenic acids conjugates showed superior cytotoxicity to both active drugs alone and their conjugates with native cyclodextrin, thus indicating that β-CD methylation can increase the cytotoxicity of the conjugates [137]. GA was subjected to a similar chemical modulation following conjugation with β-CD by using ethylene glycol linkers of variable length bearing a 1,2,3-triazole moiety; the antiviral activity of the obtained bioconjugates against the H1N1 strain in MDCK cells was subsequently evaluated and revealed six compounds with promising biological activity [138].

Currently, it is well recognized that α-CD is not able to form inclusion complexes with BA, due to the large size of the phytocompound, which cannot be accommodated inside the narrow CD cavity; however, this obstacle was overcome by the synthesis of a hexavalent conjugate of BA with α-CD using 1,2,3-triazole moieties, via a click chemistry reaction between an amydic BA derivative and the azidic α-CD derivative. The resulting bioconjugate exhibited antiviral action against the A/WSN/33 (H1N1) strain in MDCK cells, with an IC_50_ value of approximately 5.20 μM [139]. Similarly, Tian et al. synthesized bioconjugates between BA, UA and EA with α-, β- and γ-CD using 1,2,3-triazole moiety as a linker and evaluated their anti-entry effect against the HCV virus. Among the tested products, the bioconjugate of a heptavalent derivative of OA with β-CD proved to be more efficient than oseltamivir, which is generally used as a standard antiviral [140].

Although most studies approach the anticancer and/or anti-inflammatory effects of triterpenes, there are several reports regarding other biological activities that can be improved by the use of CDs. Ilexgenin A is a pentacyclic triterpene extracted from the leaves of *Ilex hainanensis* (Aquifoliaceae), which possesses several biological effects such as anti-inflammatory, anti-ischemic, antithrombotic and anti-lipidemic. The anti-lipidemic activity of ilexgenin A, quantified by the LDL and total cholesterol serum levels, was enhanced in vivo on a murine model as a result of its complexation with a highly hydrophilic β-CD polymer synthesized by using epichlorohydrin as cross-linking agent [89]; the water solubility of the inclusion complex obtained with the β-CD polymer far exceeded that of the inclusion complex formed between the phytocompound and the native β-CD, thus allowing for two to three times increased efficacy and potency compared to the pure compound. The same β-CD polymer was used to increase the solubility, bioavailability and, subsequently, the anti-inflammatory effect of pedunculoside during its in vivo assessment on a murine model [141]; the β-CD polymer exhibits advantages over the native the β-CD, such as the ability to provide multiple cavities as hosts and higher water solubility, which contributes to an optimized in vivo absorption and bioavailability.

Undoubtedly, the most explored application of CDs in the pharmaceutical field is their use as solubility enhancers in order to increase the bioavailability and therapeutical effect of numerous compounds. Despite the extensive research in the area, obstacles still exist such as the low stability of some inclusion complexes, which hampers the long-distance drug transportation in the organism or, conversely, the high stability of other inclusion complexes that could impede the release of the triterpenic compound at the target sites. Future research is needed in order to establish the most suitable CDs for triterpenes’ complexation so as to enable the use of triterpenes as therapeutic agents.

**Table 7:** In vivo anticancer activity of triterpene:cyclodextrin inclusion complexes.

Plant Extract/ Triterpenes	Cyclodextrin Type	Animal Model	Tumor Type	Tumor Cell Lines Injected	The Concentration of the Complex	Conclusion/ Observations	References
A Viscum album extract containing OA (69.4%) and BA (6.9%)	HP-β-CD	CB17/Icr-Prkdcscid/IcrCrl mice	Leukemia	NALM-6	40 mg/kg	non-significant anti-leukemic effects of the extract	[90]
		Female NOD/SCID/IL2rg mice	Leukemia	HL-60	20/40/60/mg/kg	Reduced the tumor weight	[91]
		female NOD/SCID IL2rγ null mice	Ewings Sarcoma	TC-71	40/60/ 80 mg/kg	Induced apoptosis and reduced the tumor dimension	[133]
		female NMRI-nu/nu mice					
		male C57BL/6 mice	Murine melanoma	B16.F10	$10^{6}$ cells per mouse	Reduced tumor dimension Inhibition of angiogenesis	[142]
BA	octakis-[6-deoxy-6-(2-sulfanyl ethanesulfonic acid)]- γ-cyclodextrin	8-week-old C57BL/6J female mice	Murine melanoma	B164A5	100 mg/kg	Reduced tumor dimension and weight	[81]

### 5.4. Identification of the Mechanism of Action for Triterpenic Compounds

The complexation of triterpenes with cyclodextrins can also be used in order to elucidate their mechanism of action; due to the increased bioavailability of the inclusion complexes, changes of certain biological parameters can be identified, including parameters that could not be assessed for pure triterpenes, due to their poor bioavailability. Such a study highlighted a potential mechanism of action at the mitochondrial level for Bet, following its complexation with γ-CD. The inclusion complex increased the basal respiration and oxidative phosphorylation in mitochondria isolated from senescent rats, resulting in a potential hepatoprotective effect [80].

Another study in which the complexation of triterpenes with cyclodextrins was applied in order to gain an insight into their presumable mechanism of action was conducted by Mulsow et al.; they obtained a complex aqueous extract from mistletoe, with hydrophilic (lectins, ML) and lipophilic components (OA and BA) and studied the influence of triterpenes on the penetration of ML inside several cancer cell lines, namely THP-1 (acute myelogenous leukemia), HL-60 (acute myelogenous leukemia), 143B (osteosarcoma) and TC-71 (Ewing’s sarcoma). The addition of HP-β-CD in the extraction process was essential in order to solubilize the lipophilic triterpenic acids contained in the mistletoe sprouts into the aqueous extract. Although the triterpenes did not enhance the ML uptake into the THP-1 and HL-60 cell line, in the case of the 143B cell line, ML was intracellularly detected only in the presence of triterpenes, thus emphasizing their role as a permeation enhancer. The possible underlying mechanism for this specific result may consist in the fact that, due to their lipophilic structure, triterpenes can cause structural alterations to the cell membrane, allowing for the penetration of other hydrophilic compounds into the cancer cells [143].

The inhibitory effect of MA on the aberrant crypt foci and mucin-depleted foci, two biomarkers synthesized in the early stages of colon cancer, was highlighted in a murine model in the presence of HP-β-CD, which increased MA solubility and bioavailability [144].

Another area of research in which CDs have started to be employed is the elucidation of the underlying mechanism of action of triterpenic compounds mainly due to their solubility enhancer properties. Although currently there are a limited number of studies on the subject, further research on this area could offer insightful knowledge about the molecular targets and mechanisms of triterpenic compounds that will enable the development of new therapeutic agents and prevent different interactions with other active pharmaceutical ingredients.

### 5.5. New Topical Formulations

Cyclodextrins can also be successfully used in the development of new topical treatments with superior biocompatibility following gel incorporation or the formulation of new hydrogels with polymeric supramolecular structure. Such a hydrogel was obtained after the formation of 1:1 complexes between β-CD and GA, with both components being previously attached to polymers of N,N′-dimethyl acrylamide via a free radical polymerization reaction; due to the spontaneous regeneration of the inclusion complex, the obtained hydrogel exhibits the remarkable property of spontaneous self-healing, which allows for its regeneration after mechanical destruction without the intervention of other external factors [145].

*Centanella asiatica* extract, rich in asiatic acid, madecassic acid, asiaticoside and madecassoside, was included in HP-*β*-CD in order to increase the solubility and bioavailability of the triterpenic compounds. The resulting complex was later incorporated into a gel, which was then tested on rats for the purpose of wound-healing evaluation. The tested formulations showed suitable local tolerability and stability, the lesions being completely cured within 14 days [93]; in addition, the presence of the hydrophilic CD allowed for a high content of triterpenes in the spray formulation, nearly 100% compared to the total extract. A stable gel containing asiaticoside and HP-*β*-CD was formulated; asiaticoside is a compound that promotes the synthesis of type I collagen, which is essential in curing periodontitis. The inclusion of the phytocompound inside the hydrophilic cyclodextrin favored its prolonged release into the periodontal pocket, thus improving the clinical outcome [146].

The use of CDs in the development of topical treatments has led to formulations with possible use in wound-healing management that possess improved bioavailability, prolonged release of the active ingredients and high biocompatibility. A possible limitation of this type of formulation that remains to be further explored and overcome is the reduced stability that could significantly reduce the shelf life of these formulations.

### 5.6. Other Applications

*Ganoderma lucidum* is a mushroom of the Ganodermataceae family used in traditional Chinese medicine for its antitumor, anti-inflammatory, anti-hepatotoxic, antioxidant and immunomodulatory properties. Studies have shown that the spores of this species contain the highest concentration of active compounds, including ganoderic acid A; however, the spores possess an outer hard shell, called sporoderm, which prevents their exterior release. In order to overcome this obstacle, Wang et al. employed a chemical process using substances such as sodium hydroxide, urea and alcohol, followed by ultrasonication and refrigeration to create pores into the sporoderm. Subsequently, β-CD was used in the post-processing stage to seal the pores formed after the removal of the reagents used in the chemical process. β-CD has fulfilled three roles in this process: (1) forms inclusion complexes with the bioactive compounds, thus improving their solubility and stability; (2) seals the holes of the sporoderm, thus ensuring the preservation of the active compounds contained inside the spores against volatilization and oxidation; and (3) modifies the surface of the spores, thus rendering them easily dispersible in water, a significant step in human digestion; thereby, the method works effectively in achieving highly bioactive and stable final product [147].

CDs can be also used to reduce the unpleasant organoleptic characteristics of certain active compounds. One such study was conducted in order to reduce the bitterness of the fruit juice of *Momordica charantia* (Cucurbitaceae), which contains two triterpenic saponins, momordicoside K and momordicoside L, without reducing its antihyperglycemic effect; β-CD was used as a host molecule for the bitter compounds. The incorporation of the juice into β-CD not only decreased its bitterness, but also determined a small decrease of its antihyperglycemic effect, as reflected by the inhibition of α-glucosidase test; however, by improving its organoleptic properties, it is possible to increase the tolerability and use of the *Momordica* juice as a complementary treatment in diabetes [94]. In a similar manner, by complexation with β-CD and γ-CD, the sweet taste of GA was reduced. The sweet taste, which can be unpleasant for some people, is attributed to the formation of aggregates between GA molecules and the sweet taste receptors situated on the tongue surface [148].

In this subsection, the application of CDs in improving the preservability and organoleptic properties of certain extracts containing triterpenic compounds or pure compounds such as GA has been highlighted. More information about the stability of the obtained formulations and complexes is required in order to enable their use in the pharmaceutical and food industry.

## 6. Conclusion and Future Perspectives

Although this year marks 130 years since the discovery of CDs by Villiers, they remain compounds of high value due to their remarkable physicochemical properties, which enable their use in various domains such as food, cosmetic and pharmaceutical development. The capacity to incorporate inside their internal cavity guests with lipophilic properties or to form bioconjugates through derivatization of the outer surface hydroxylic groups triggered their extensive use in drug design, especially in the development of phytochemical-based drugs. Triterpenes are phytocompounds that possess remarkable biological activities and represent one of the most extensively studied class of natural compounds for the development of new effective drugs. The inclusion complexes and bioconjugates obtained between triterpenic compounds and CDs expanded our goals towards the identification of the underlying mechanism of action of triterpenes and the development of environment-friendly bioproduction methods using microorganisms and algae cells as biofactories, new effective isolation and separation protocols from plant sources and the design of potential new drugs and topical formulations with improved bioavailability and activity. Nevertheless, some of the research areas mentioned in this paper, such as bioproduction of triterpenic compounds, are still in the early stages of development and need to identify methods by which these techniques could be used on a larger scale; even the more established research areas need to overcome obstacles such as the low stability of the resulting inclusion complexes.

## Figures and Tables

**Table 1:** Biological activities of triterpenic compounds.

Number	Chemical Structure	Triterpenic Compound	Biological Effects	References
1	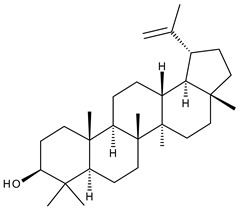	Lupeol (lup-20(29)-en-3-ol)	anticancer, antihyperglycemic, antidyslipidemic, anti-inflammatory, antioxidant, antimicrobial	[18,19]
2	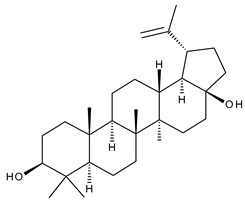	Betulin (Bet, 3-lup-20(29)-ene-3β,28-diol)	anti-inflammatory, antimicrobial, antifibrotic, antiproliferative, wound-healing properties	[20,21,22,23]
3	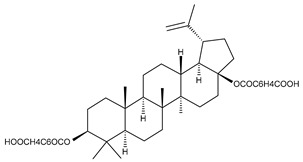	betulin 3,28-diphthalate (DPhB)	no data available	[24]
4	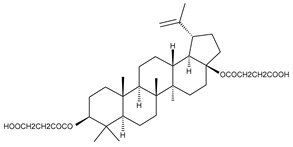	betulin 3,28-disuccinate (DScB)	no data available	[24]
5	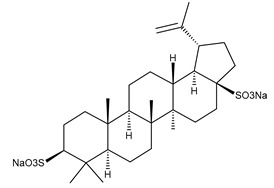	betulin 3,28-disulfate (DSB)	no data available	[25]
6	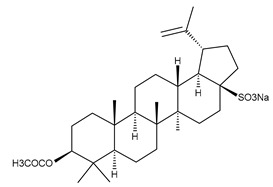	betulin 3-acetate-28-sulfate (ASB)	no data available	
7	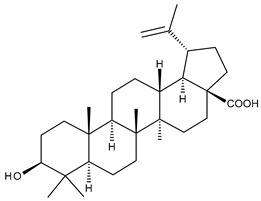	Betulinic acid (BA, 3β-hydroxy-lup-20(29)-en-28-oic acid)	antiviral, antihyperglycemic, anti-inflammatory, antioxidant, anticancer	[26,27,28,29,30,31,32]
8	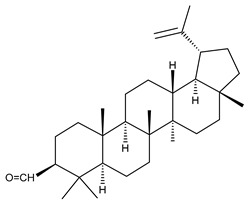	Betulonic acid (BoA, lup-20(29)-en-3-oxo-28-oic)	anticancer, antiviral, antimicrobial	[33,34]
9	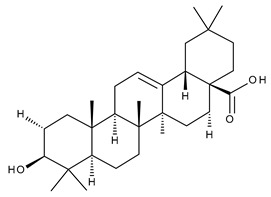	Oleanolic acid (OA, 3β-hydroxyolean-12-en-28-oic acid)	antioxidant, anticancer, antidiabetic, antihypertensive, hepatoprotective, antimicrobial, antiparasitic	[35]
10	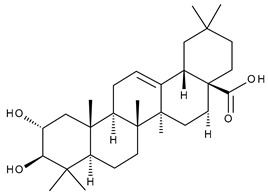	Maslinic acid (MA, (2α,3β)-2,3-dihydroxyolean-12-en-28-oic acid)	antioxidant, antitumor, neuroprotective, antidiabetic, cardioprotective, antiparasitic	[36]
11	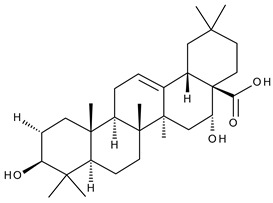	Echinoscystic acid (EA, 3,16-dihydroxyolean-12-en-28-oic acid)	analgesic, anti-inflammatory, neuroprotective, antiosteoporotic	[37,38,39,40]
12	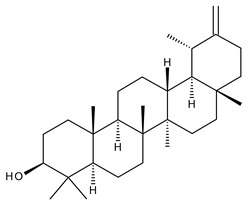	Taraxasterol ((3β,18α,19α)-Urs-20(30)-en-3-ol)	anticancer, anti-inflammatory, antimicrobial, effective against snake venom	[41]
13	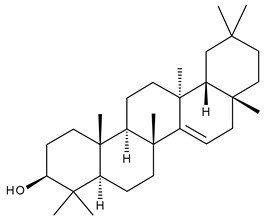	Taraxerol (3β-Taraxerol)		
14	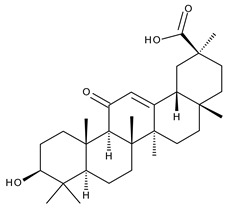	Glycyrrhetinic acid (GA, 3beta-Hydroxy-11-oxoolean-12-en-30-oic acid)	anti-inflammatory, anticancer	[42,43]
15	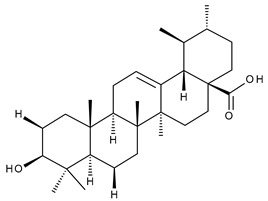	Ursolic acid (UA, 3 β-hydroxy-urs-12-en-28-oic acid)	anticancer, antihypertensive, reduces kidney damage, anti-HIV, antihepatitic, antimalarial, antibacterial	[44]
16	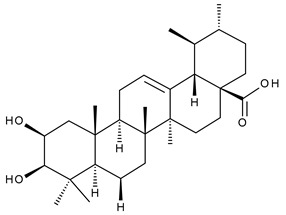	Corosolic acid (CA, (2α,3β)-2,3-Dihydroxyurs-12-en-28-oic acid)	antihyperglycemic, anticancer	[45,46,47,48,49]
17	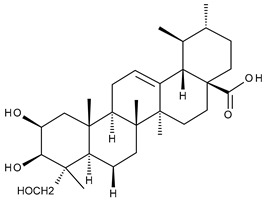	Asiatic acid ((2α,3β)-2,3,23-Trihydroxyurs-12-en-28-oic acid)	anticancer, neuroprotective, antihypertension, anti-atherosclerotic and wound-healing properties	[50]
18	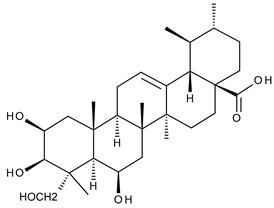	Madecassic acid ((2α,3β,6β)-2,3,6,23-Tetrahydroxyurs-12-en-28-oic acid)	anti-ischemic in retinopathies	[51]
19	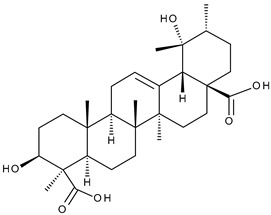	Ilexgenin A ((3β)-3,19-Dihydroxyurs-12-ene-24,28-dioic acid)	anti-atherosclerotic, anti-inflammatory, anticancer	[52,53,54,55,56]
20	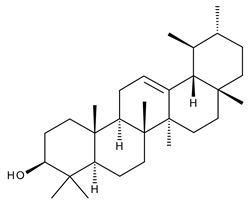	α-Amirin	analgesic, anticonvulsant, anticholytic, antidepressive, anti-inflammatory, antihyperglycemic, gastroprotective, hepatoprotective, hypolipidemic	[57]
21	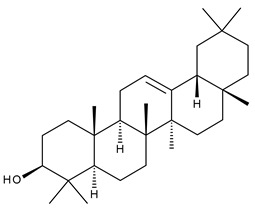	β-Amirin	analgesic, anticonvulsant, anticholytic, antidepressive, anti-inflammatory, antihyperglycemic, gastroprotective, hepatoprotective, hypolipidemic	
22	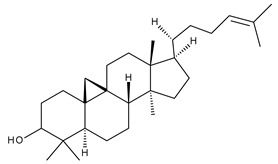	Cycloartenol ((3β,9β)-9,19-Cyclolanost-24-en-3-ol)	anti-inflammatory, anticancer, antioxidant, antimicrobial	[58]
23	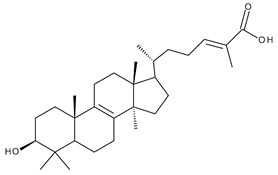	GAN-HLDOA (ganoderic acid 3-hydroxy-lanosta-8,24-dien-26-oic acid)	anticancer	[59]

**Table 2:** Physicochemical and toxicological properties of CDs.

Characteristic	Type of Native Cyclodextrins			Type of Semisynthetic Cyclodextrins		
	α-CD	β-CD	γ-CD	HP-β-CD	Methyl-β-CD	DM-β-CD
Number of glucopyranose units	6	7	8	7	7	7
Molecular weight (Da)	972	1135	1297	1541	1303	1331
Solubility in water (g/100 mL) at 25 °C	14.5	1.85	23.2	>60	>50	no data available
Internal diameter (nm)	0.47–0.53	0.60–0.65	0.75–0.83	no data available	no data available	no data available
External diameter (nm)	1.46	1.54	1.75	no data available	no data available	no data available
Toxicological properties	no toxic effects reported	nephrotoxicity (parental administration)	no toxic effects reported	low hemolytic effect	cholesterol depleting property	cholesterol depleting property
Reference	[70]	[63]	[71]	[72,73]	[72,74]	[75]

**Table 3:** Methods of preparation for cyclodextrin inclusion complexes with triterpenic compounds.

Triterpenic Compund	Cyclodextrin Type	Method of Preparation	State of Aggregation of the Inclusion Complex	Solvent Composition	References
Bet	γ-CD	Kneading	liquid	EtOH:water (1:1 *v*/*v*)	[80]
BA	γ-CD	Kneading	liquid	EtOH:water (1:1 *v*/*v*)	[80]
BA	octakis-[6-deoxy-6-(2-sulfanyl ethanesulfonic acid)]- γ-CD	Kneading	liquid	EtOH:water (1:1 *v*/*v*)	[81]
BA	β-CD	Co-precipitation	liquid	β-CD in deionized water and BA in MeOH	[82]
OA	α-CD, β-CD γ-CD, HP-α-CD, HP-β-CD, HP-γ-CD	Kneading	liquid	water	[83]
OA	amino-appended β-CDs	Slurry-complexation	liquid	water	[84]
MA	α-CD, β-CD γ-CD, HP-α-CD, HP-β-CD, HP-γ-CD	Kneading	liquid	water	[83]
UA	HP-β-CD, HP-γ-CD	Kneading	liquid	EtOH:water (1:1 *v*/*v*)	[85,86]
OA	HP-β-CD, HP-γ-CD	Kneading	liquid	EtOH:water (1:1 *v*/*v*)	[85,86]
Lupeol	β-CD	Spray-drying	liquid	EtOH:water (1:2 *v*/*v*)	[61]
α,β-amyrin	β-CD, HP-β-CD	Physical mixing	solid	-	[87]
		Kneading	liquid	water and acetone	
Koetjapic acid	HP-β-CD (1:2, 1:4, 1:6)	Kneading	liquid	water	[88]
ilexgenin A	β-CD polymer (β-CD units were linked using epichlorohydrin)	Slurry-complexation	liquid	water	[89]

**Table 4:** Methods of preparation of cyclodextrin inclusion complexes with plant extracts containing triterpenic compounds.

Plant Species	Triterpenes in the Extract	Cyclodextrin Type	Method of Complex Formulation	Solvent Composition	Observations	References
Viscum album	OA and BA	HP-β-CD	Physical mixing	-		[90,91]
Viscum album	OA and BA	HP-β-CD	Physical mixing	-		[92]
Centella asiatica	asiatic acid, madecassic acid, asiaticoside, madecassoside	HP-β-CD	Kneading	water		[93]
Momordica Charantia	momordico-side K and momordico-side L	β-CD	Kneading	Juice of Momordica Charantia		[94]
Boswellia serrata	Boswellic acids	HP-β-CD	Kneading	EtOH:water (1:1 *v*/*v*)		[95]
			Co-precipitation	Boswellia extract in EtOH and HP-β-CD in EtOH:water (1:1 *v*/*v*)		
			Solvent evaporation	Boswellia extract in EtOH and HP-β-CD in EtOH:water (1:1 *v*/*v*)	Stirring 500 rpm for 24 h at room temperature	

**Table 5:** Kc values of inclusion complexes between HP-γ-CD and Bet derivatives.

Triterpenic Compounds	Cyclodextrin Type	log (Kc), 25 °C	Reference
Triterpenes			
Bet	HP-γ-CD	3.82 ± 0.12	[99]
Triterpenoids			
DPhB	HP-γ-CD	7.23 ± 0.03	[24]
DScB		7.13 ± 0.10	
DSB		6.70 ± 0.05	[25]
ASB		7.03 ± 0.10	

## Data Availability

Not applicable.
